# The association between soluble klotho and cardiovascular parameters in chronic kidney disease: results from the KNOW-CKD study

**DOI:** 10.1186/s12882-018-0851-3

**Published:** 2018-03-05

**Authors:** Hyo Jin Kim, Eunjeong Kang, Yun Kyu Oh, Yeong Hoon Kim, Seung Hyeok Han, Tae Hyun Yoo, Dong-Wan Chae, Joongyub Lee, Curie Ahn, Kook-Hwan Oh

**Affiliations:** 1 Department of Internal Medicine, Dongguk University College of Medicine, Gyeongju-si, Gyeongsangbuk-do South Korea; 2 Department of Internal Medicine, Seoul National University College of Medicine, Seoul, Korea; 3 Department of Internal Medicine, Inje University, Busan Paik Hospital, Busan, South Korea; 4 Department of Internal Medicine, Yonsei University College of Medicine, Seoul, South Korea; 5 Medical Research Collaborating Center, Seoul National University Hospital and Seoul, National University College of Medicine, Seoul, South Korea

**Keywords:** Serum klotho, Soluble klotho, Chronic kidney disease, Left ventricular mass index, Left ventricular hypertrophy, Pulse wave velocity

## Abstract

**Background:**

Klotho, a protein linked to aging, has emerged as a pivotal player in mineral bone metabolism and might explain the relationship between chronic kidney disease (CKD) and cardiovascular disease (CVD). The present study aimed to investigate the association between serum klotho and cardiac parameters from a large-scale Korean CKD cohort.

**Methods:**

We analyzed 2101 participants from KoreaN Cohort Study for Outcome in Patients With Chronic Kidney Disease (KNOW-CKD) cohort who had been measured for serum klotho levels. Left ventricular hypertrophy evaluated by left ventricular mass index (LVMI) and arterial stiffness measured by brachial-to-ankle pulse wave velocity (baPWV) were explored as cardiovascular parameters.

**Results:**

Patients were 53.6 ± 12.2 years old and 61.1% were male. The mean estimated glomerular filtration rate (eGFR) was 53.0 ± 30.7 mL/min/1.73m^2^. The median serum klotho level was 536 (interquartile range [IQR]: 420–667) pg/mL. Advanced CKD stages were associated with lower serum klotho levels (*P* < 0.001, *P* for linear trend < 0.001). Ascending quartiles of klotho were significantly associated with decreased LMVI (*P* < 0.001, *P* for linear trend< 0.001). A multivariable linear regression model showed serum klotho had a significant inverse association with LVMI (*β* − 0.04; 95% CI [confidence interval] -0.004, − 0.00007; *P* = 0.041). However, there was no significant association between serum klotho and baPWV after adjustment (*β* 0.003; 95% CI -0.04, 0.05; *P* = 0.876).

**Trial registration:**

This trial was registered on ClinicalTrials.gov on 28 June 2012 (NCT01630486).

**Conclusions:**

Serum klotho was an independent biomarker of LVMI, but not arterial stiffness.

## Background

Patients with chronic kidney disease (CKD) present with a higher burden of cardiovascular disease (CVD) and cardiovascular mortality than the general population [1, 2]. Left ventricular myocardial hypertrophy, the most commonly diagnosed cardiovascular abnormality in CKD patients, is secondary to both volume and pressure overload [3]. Cardiac hypertrophy is an important cause of cardiovascular morbidity and mortality for CKD patients because it can lead to congestive heart failure, arrhythmia, ischemic cardiomyopathy, even in the absence of coronary artery disease, and sudden death in CKD patients [4–6]. Arterial stiffness in CKD patients caused by arteriosclerosis with thickening and stiffening of the arterial wall [7] brings about cardiac hypertrophy and a negative prognostic value for CVD [8, 9].

Mineral bone metabolism is important in CKD, and progressive deterioration of calcium-phosphorus homeostasis is associated with cardiovascular complications. Impaired calcium-phosphorus homeostasis can cause cardiac hypertrophy and vascular calcification. Klotho has emerged as a pivotal player in calcium-phosphorus homeostasis and mineral metabolism regulation in CKD, and it might explain the relationship between CKD and CVD. The *Klotho* gene, which was originally identified as an aging suppressor gene, is closely associated with CKD. In a previous study, klotho knock-out mice had similarities with CKD patients, such as hyperphosphatemia, ectopic soft tissue calcification, and arteriosclerosis [10], suggesting that CKD might result from a state of klotho deficiency. Thus, in addition to serving as a biomarker for CKD, klotho deficiency is also a pathogenetic indicator for both renal and extra-renal complications in CKD [11]. In previous experimental studies, restoration of serum klotho levels ameliorated cardiac hypertrophy and vascular calcification [12, 13], and haplodeficiency of the *Klotho* gene caused arterial stiffness [14]. Clinical data supporting the above experimental studies are scarce and present mixed results [15]. Yang et al. [16] showed that cardiac hypertrophy evaluated by left ventricular mass index (LVMI) was negatively associated with serum klotho in 86 CKD patients. They did not show the association between klotho and LVMI for CKD patients in an adjustment model. In another study, there was no significant association between serum klotho and LVMI in dialysis patients [17]. Previous studies have been conducted on a small number of patients and few studies have focused on CKD patients for the association between klotho and LVMI. Given the negative effects of klotho deficiency, its associated cardiovascular complications in preclinical studies, and the limited number of clinical studies, the current study aimed to investigate the association between serum klotho and cardiovascular parameters in CKD patients, using the baseline cross-sectional data set of a large-scale Korean CKD cohort.

## Methods

### Study population

The KoreaN Cohort Study for Outcome in Patients With Chronic Kidney Disease (KNOW-CKD) was a Korean multicenter prospective cohort study that enrolled participants with CKD from stage 1 to 5 (predialysis) from nine clinical centers of major university-affiliated hospitals in Korea. Details about the study design and methods are described elsewhere [18]. Subjects with severe heart failure (New York Heart Association Class III or IV) were excluded from enrollment according to study protocol. Among the 2238 participants enrolled in the KNOW-CKD study from June2011 to January2016, 2113 individuals had their serum klotho levels assayed. Twelve patients were excluded as extreme values (serum klotho either lower than detectable range or above 6000 pg/mL). These values were not influence value by Cook’s distance analysis. Finally 2101individualswere included in the cross-sectional analysis. The study protocol was approved by the ethical committee of each participating clinical center, including the Institutional Review Boards of Seoul National University Hospital, Severance Hospital, Kangbuk Samsung Medical Center, Seoul St. Mary’s Hospital, Gil Hospital, Eulji General Hospital, Chonnam National University Hospital, and Busan Paik Hospital. All participating patients provided written informed consent. The study protocol was in accordance with the principles of the Declaration of Helsinki.

### Clinical data collection and laboratory analyses

Baseline demographic characteristics and laboratory values at enrollment were extracted from an electronic data management system (http://www.phactaX.org). Serum creatinine was measured by an isotope dilution mass spectrometry (IDMS)-traceable method [19] at a central laboratory. The estimated glomerular filtration rate (eGFR) was estimated using the Chronic Kidney Disease Epidemiology Collaboration (CKD-EPI) creatinine eq. [20].The serum α-klotho level was measured using an enzyme-linked immunosorbent assay (ELISA) kit (Immuno-Biological Laboratories Co., Gunma, Japan) according to the manufacturer’s protocol l [21]. The intra-assay and inter-assay coefficients of variation were 2.7–3.5% (Klotho levels186.64–2968.78 pg/mL) and 2.9–11.4% (Klotho levels 165.47–2903.01 pg/mL), respectively. The data for intra-assay and inter-assay coefficients of variations were confirmed in our central laboratory by measurements of a serum control in 20 repeats on each ELISA plate. The intra-assay and inter-assay coefficients of variations were 0.57–1.78% and 3.01–6.12% (Klotho levels 120.30–4468.50 pg/mL), respectively. The standard curve was found to be linear up to 4000 pg/mL. C-terminal FGF23 was measured using second generation human FGF23 ELISA kit (Immutopics, San Clemente, California, USA) according to the manufacturer’s protocol. The intra-assay and inter-assay coefficients of variation as reported by the manufacturer were 1.4–2.4% (FGF23 levels 33.7–302 RU/mL) and 2.4–4.7% (FGF23 levels 33.6–293 RU/mL), respectively.

### Cardiovascular parameters

Cardiovascular parameters were evaluated using left ventricular mass index (LVMI) and pulse wave velocity (PWV), which represent left ventricular hypertrophy and arterial stiffness, respectively. Two-dimensional echocardiography was conducted by experts at each hospital, and the LVMI was calculated by dividing the left ventricular (LV) mass by theheight^2.7^ [22]. LVMI was calculated in the cohort center using the same formula. LV mass was calculated by the formula 0.8 x{1.04×[(LVIDd +PWTd + SWTd)^3^ - LVIDd^3^]} + 0.6 (g), where LVIDd, PWTd, and SWTd are LV internal diameter at end diastole, posterior wall thickness at end diastole, and septal wall thickness at end diastole, respectively [23]. Left ventricular hypertrophy (LVH) was defined as LVMI ≥50 g/m^2.7^ in men and ≥47 g/m^2.7^in women [24]. Relative wall thickness (RWT) was calculated to as two times posterior wall thickness/LV internal linear dimension in diastole (RWT = [2 x PWTd]/LVIDd). RWT was considered to be increased if > 0.42. LVMI and RWT were used to categorize LV geometry: normal (normal LVMI and normal RWT), concentric remodeling (normal LVMI and RWT > 0.42), concentric LVH (increased LVMI and RWT > 0.42), eccentric LVH (increased LVMI and RWT ≤ 0.42). Systolic heart dysfunction and diastolic heart dysfunction were defined as an LV ejection fraction < 50% and a ratio (E/E’ ratio) of mitral peak velocity of early filling (E) to early diastolic mitral annular velocity(E’) > 15 on echocardiography, respectively [25, 26].Arterial stiffness was measured with brachial-to-ankle PWV (baPWV) and heart-to-femoral PWV (hfPWV) [27]. Among the nine participating centers, hfPWV was measured only at five centers where the equipment was available. hfPWV represented central arterial stiffness, while baPWV represented peripheral arterial stiffness. The abdominal aorta calcification(AAC) score was measured with simple lateral lumbar radiography with a range of 0 to 24 [28]. The coronary artery calcium score (CACS) was measured by computed tomography and was presented as the Agatson score [29, 30].

### Statistical analyses

Categorical variables were evaluated using the Chi-square test and presented as frequencies and percentages. Continuous variables were analyzed with one-way analysis of variance (ANOVA) or the Kruskal-Wallis test. The Kolmogorov-Smirnov test was used to analyze the normality of the distribution of parameters. Results are presented as the mean ± standard deviation (SD) for normally distributed variables and the median (interquartile range [IQR]) for variables with skewed distribution. Participants were classified into quartiles according to their serum klotho level. We analyzed the serum klotho value across the CKD stages by Kruskall-Wallis test and *P* value is the difference between CKD stages. *P*-for trend of klotho level with advanced CKD stages was measured by Jonckheere-Terpstra test (Jonckheere trend test). We analyzed LVMI and PWV across the klotho quartiles by ANOVA and *P* value is the difference between klotho quartiles. *P*-for trend of LMVI and PWV value with higher klotho quartiles was measured using ANOVA trend analyses using polynomial contrasts. We employed a multivariable linear regression model analysis with adjustment (enter method) to investigate the associations of left ventricular hypertrophy and PWV with serum klotho level. LVMI and PWV are continuous variables. LMVI and PWV (dependent variables) corresponding to the independent variables fulfilled the assumptions for linear regression model: normality, homoscedasticity, independence, and linearity. For all linear regression models, variables were selected based on the prior studies and physiological reasoning [31–33]. Collinearity among variables was tested. *P-*values < 0.05 were considered statistically significant. The SPSS statistical software (SPSS version 18.0, Chicago, IL, USA) was used for all descriptive and outcome analyses.

## Results

### Demographic and baseline clinical characteristics of participants

**Fig. 1:**
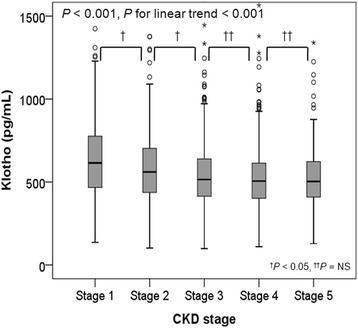
Serum klotho levels across CKD stages. Advanced CKD stages were associated with lower serum klotho levels (*P* < 0.001, *P* for linear trend < 0.001). NS, non significant

The clinical characteristics of the study patients at enrollment are shown in Table 1. Among 2101 patients, the mean age was 53.6 ± 12.2 years, and 61.1% were male. The mean eGFR was 53.0 ± 30.7 mL/min/m^2^. Patients with diabetes mellitus (DM) and hypertension (HTN) comprised 33.9% and 96.1% of the subjects, respectively. The median serum klotho level was 536 (interquartile range [IQR]:420–667) pg/mL. Serum klotho levels according to CKD stages are shown in Fig. 1. Advanced CKD stages were associated with lower serum klotho levels (*P* < 0.001, *P* for linear trend< 0.001).

**Table 1:** The clinical characteristics of the study subjects at enrollment stratified by serum klotho level

Characteristics	Total (*N* = 2101)	Klotho groups				*P*-value	*P* for trend
		1st quartile (*n* = 524) (99–419 pg/mL)	2nd quartile (*n* = 528) (420–536 pg/mL)	3rd quartile (*n* = 523) (537–666 pg/mL)	4th quartile (*n* = 526) (667–3641 pg/mL)		
Age (mean ± SD)	53.6 ± 12.2	54.0 ± 11.8	54.6 ± 11.9	53.5 ± 12.2	52.2 ± 12.5	0.015	0.007
Gender, male, n (%)	1284 (61.1)	330 (63.0)	319 (60.4)	317 (60.6)	318 (60.5)	0.795	0.440
BMI (kg/m^2^)	24.5 ± 3.4	24.8 ± 3.5	24.6 ± 3.2	24.5 ± 3.4	24.2 ± 3.3	0.036	0.005
DM, n (%)	712 (33.9)	171 (32.6)	192 (36.4)	172 (32.9)	177 (33.7)	0.560	0.962
HTN, n (%)	2020 (96.1)	507 (96.8)	517 (97.9)	501 (95.8)	495 (94.1)	0.011	0.007
SBP (mmHg)	128.5 ± 16.4	129.3 ± 16.1	128.5 ± 16.6	127.9 ± 16.9	128.5 ± 16.1	0.599	0.370
DBP (mmHg)	76.9 ± 11.2	76.7 ± 11.8	77.0 ± 11.1	76.6 ± 11.2	77.2 ± 10.5	0.786	0.667
ACEi or ARB, yes, n (%)	1795 (85.6)	458 (87.4)	461 (87.5)	442 (84.7)	434 (82.7)	0.075	0.013
eGFR (ml/min/1.73m^2^)	53.0 ± 30.7	48.2 ± 28.6	47.6 ± 27.8	54.6 ± 31.6	61.7 ± 32.5	< 0.001	< 0.001
Hemoglobin (g/dL)	12.8 ± 2.0	12.6 ± 1.9	12.6 ± 2.0	12.9 ± 2.0	13.3 ± 2.1	0.001	< 0.001
Uric acid (mg/dL)	7.0 ± 1.9	7.4 ± 2.0	7.2 ± 1.9	6.9 ± 1.9	6.6 ± 1.8	< 0.001	< 0.001
Albumin (g/dL)	4.2 ± 0.4	4.1 ± 0.4	4.2 ± 0.4	4.2 ± 0.4	4.2 ± 0.5	0.316	0.068
Total cholesterol (mg/dL)	174.3 ± 39.4	174.0 ± 39.3	172.0 ± 38.0	172.5 ± 40.3	178.8 ± 39.6	0.021	0.053
CRP, median, (Q1, Q3) (mg/dL)	0.06 (0.02, 0.17)	0.08 (0.03, 0.20)	0.06 (0.03, 0.16)	0.06 (0.02, 0.16)	0.05 (0.02, 0.13)	< 0.001	< 0.001
Phosphorus (mg/dL)	3.7 ± 0.7	3.8 ± 0.7	3.7 ± 0.7	3.7 ± 0.7	3.6 ± 0.6	0.004	0.001
Corrected Ca (mg/dL)	9.0 ± 0.4	9.0 ± 0.4	9.0 ± 0.5	9.0 ± 0.4	8.9 ± 0.4	0.505	0.530
Klotho, median (Q1, Q3) (pg/mL)	536 (420, 667)	335 (269, 383)	479 (449, 505)	593 (562, 626)	788 (714, 913)	< 0.001	< 0.001
25(OH)VitD, median (Q1, Q3) (ng/mL)	16.5 (12.7, 21.3)	16.7(13.5, 21.7)	16.6(13.1, 21.2)	16.4(12.6, 21.3)	16.1(12.1, 21.0)	0.040	0.005
1,25(OH)_2_VitD, median (Q1, Q3) (pg/mL)	25.4 (20.1, 33.7)	26.4 (20.1, 36.2)	24.6 (19.1, 31.6)	24.3 (19.4, 32.5)	26.6 (21.4, 34.4)	< 0.001	0.599
iPTH, median (Q1, Q3) (pg/mL)	51.5 (33.2, 84.0)	55.0 (35.0, 86.1)	53.0 (34.5, 88.8)	52.6 (33.9, 86.8)	46.3 (30.2, 74.8)	0.005	0.003
C-terminal FGF23, median (Q1, Q3) (RU/mL)	17.9 (0.4, 31.3)	18.5 (0.3, 30.2)	19.0 (1.0, 32.3)	18.6 (0.8, 31.5)	10.4 (0.2, 31.4)	0.094	0.419
UACR, > 300 mg/g, n (%)	884 (49.9)	217 (52.3)	255 (56.0)	205 (45.5)	207 (45.9)	< 0.001	< 0.001
LVMI, median (Q1, Q3) (g/m^2.7^)	40.3 (33.8, 48.6)	41.3 (34.7, 50.2)	40.9 (34.4, 49.2)	39.5 (33.5, 49.3)	39.6 (32.5, 46.7)	< 0.001	< 0.001
LVH, n (%)	509 (24.8)	145 (28.3)	139 (27.0)	128 (24.8)	97 (19.2)	0.004	0.001
LV geometry, n (%)						0.084	0.006
Normal	1248 (60.9)	303 (59.1)	304 (59.0)	316 (61.2)	325 (64.2)		
Concentric remodeling	293 (14.3)	65 (12.7)	72 (14.0)	72 (14.0)	84 (16.6)		
Eccentric LVH	254 (12.4)	76 (14.8)	70 (13.6)	60 (11.6)	48 (9.5)		
Concentric LVH	255 (12.4)	69 (13.5)	69 (13.4)	68 (13.2)	49 (9.7)		
baPWV (cm/s)	1534 ± 344	1552 ± 348	1552 ± 322	1527 ± 357	1507 ± 347	0.127	0.024
^a^hfPWV (cm/s)	1018 ± 274	1053 ± 300	1028 ± 257	1005 ± 277	989 ± 258	0.022	0.002
AAC ≥ 1, n (%)	703 (35.0)	172 (35.0)	194 (38.0)	183 (36.6)	154 (30.6)	0.079	0.127
CACS > 100, n (%)	498 (24.6)	133 (26.2)	131 (25.7)	121 (24.0)	113 (22.6)	0.528	0.143
LVEF < 50%	30 (1.5)	8 (1.5)	5 (1.0)	7 (1.3)	10 (2.0)	0.607	0.497
E/E’ > 15	176 (8.6)	46 (9.0)	47 (9.1)	42 (8.2)	41 (8.1)	0.923	0.536

^a^1243 patients measured hfPWV (vs. 1907 patients measured baPWV) at the study enrollment

SD, standard deviation; BMI, body mass index; DM, diabetes mellitus; HTN, hypertension; SBP, systolic blood pressure; DBP, diastolic blood pressure; eGFR, estimated glomerular filtration rate by CKD-EPI creatinine equation; CRP, C-reactive protein; Ca, calcium; 25(OH)VitD, 25-hydroxy vitamin D; 1,25(OH)_2_VitD, 1,25-hydroxy vitamin D; iPTH, intact parathyroid hormone; FGF23, fibroblast growth factor 23; UACR, random urine albumin-creatinine ratio; ACEi, angiotensin converting enzyme inhibitor; ARB, angiotensin receptor blocker; LVMI, left ventricular mass index; LVH, left ventricular hypertrophy; baPWV, brachial-to-ankle pulse wave velocity; hfPWV, heart-to-femoral pulse wave velocity; AAC, Abdominal aorta calcification; CACS, coronary artery calcium score; LVEF, left ventricular ejection fraction; E/E’, ratio of mitral peak velocity of early filling (E) to early diastolic mitral annular velocity (E’)

### Comparison of baseline characteristics according to serum klotho levels

We compared baseline characteristics classified by the klotho quartiles (Table 1). Patients in the highest (4th) quartile group were younger and had a lower body mass index (BMI). eGFR and hemoglobin levels were higher in the 4th quartile klotho group. Uric acid, C-reactive protein (CRP), phosphorus, and intact parathyroid hormone(iPTH) were lower in the 4th quartile klotho group.

### Serum klotho and cardiovascular parameters

**Fig. 2:**
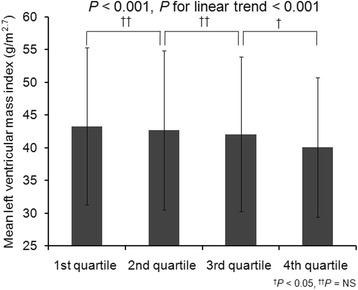
Mean left ventricular mass index across the quartiles of klotho. Ascending quartiles of klotho were significantly associated with decreased left ventricular mass index (*P* < 0.001, *P* for linear trend < 0.001). NS, non significant

**Fig. 3:**
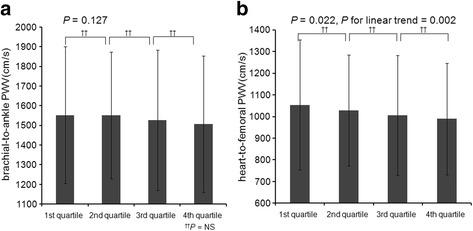
Mean pulse wave velocity across the quartiles of klotho. **a** Brachial-to-ankle pulse wave velocity had a tendency of decreasing across the quartiles of klotho. **b** Ascending quartiles of klotho were significantly associated with decreased heart-to-femoral PWV (*P* = 0.022, *P* for linear trend = 0.002). PWV, pulse wave velocity; NS, non significant

Ascending quartiles of klotho were significantly associated with decreased LMVI (*P* < 0.001, *P* for linear trend< 0.001; Fig. 2). A total of 509 (24.8%) patients had LVH (281 [22.4%] in male, 228 [28.6%] in female). LV geometry was not significantly different among the klotho quartile groups (*P* = 0.084). Table 2 summarizes the results of the multivariable linear regression analysis of the association between klotho and LVMI. After adjustment for age, gender, DM, HTN, BMI, systolic blood pressure, eGFR, hemoglobin, phosphorus, corrected calcium, and FGF23, serum klotho had a significant inverse association with LVMI (*β* - 0.04; 95% CI -0.004, − 0.00007; *P* = 0.041; Table 2). baPWV had a tendency of decreasing across the quartiles of klotho but it is not statistically significant (*P* = 0.127; Fig. 3A).Ascending quartiles of klotho were significantly associated with decreased hfPWV (*P* = 0.022, *P* for linear trend = 0.002; Fig. 3B). However, there was no significant association of serum klotho with baPWV (*β* 0.003; 95% CI -0.04, 0.05; *P* = 0.876) and hfPWV (*β* − 0.013; 95% CI -0.06, 0.034; *P* = 0.564) after adjustment (Table 3). AAC and CACS values were not significantly different among the klotho groups (Table 1). Systolic or diastolic heart function values as LVEF and E/E’ were not significantly different among the klotho groups (Table 1).

**Table 2:** Multivariable linear regression analysis presenting associations between klotho and left ventricular mass index

Variable	Model A		Model B		Model C	
	*β* (95% CI)	*P*- value	*β* (95% CI)	*P*- value	*β* (95% CI)	*P*- value
Klotho (pg/mL)	− 0.09 (− 0.006, − 0.002)	< 0.001	−0.079 (− 0.005, − 0.002)	< 0.001	−0.04 (− 0.004, − 0.00007)	0.041

Model A: Adjusted for klotho, age and gender

Model B: Adjusted for klotho, age, gender, DM, HTN, BMI, and SBP

Model C: Adjusted for klotho, age, gender, DM, HTN, BMI, SBP, eGFR, hemoblogin, phosphorus, corrected Ca, and FGF23

*β*, Standardized coefficient; CI, confidence interval; DM, diabetes mellitus; HTN, hypertension; BMI, body mass index; SBP, systolic blood pressure; eGFR, estimated glomerular filtration rate by CKD-EPI creatinine equation; Ca, calcium; FGF23, fibroblast growth factor 23

**Table 3:** Multivariable linear regression analysis presenting associations between klotho and pulse wave velocity

	Variable	Model A		Model B		Model C	
		*β* (95% CI)	*P*- value	*β* (95% CI)	*P*- value	*β* (95% CI)	*P*- value
baPWV	Klotho (pg/mL)	−0.011 (− 0.07, 0.04)	0.582	−0.017 (− 0.07, 0.02)	0.329	0.003 (− 0.04, 0.05)	0.876
hfPWV	Klotho (pg/mL)	−0.029 (− 0.09, 0.02)	0.228	− 0.037 (− 0.09, 0.01)	0.087	−0.013 (− 0.06, 0.034)	0.564

Model A: Adjusted for klotho, age and gender

Model B: Adjusted for klotho, age, gender, DM, HTN, BMI, and SBP

Model C: Adjusted forklotho, age, gender, DM, HTN, BMI, SBP, eGFR, hemoblogin, phosphorus, corrected Ca, and FGF23

*β* Standardized coefficient, *CI* confidence interval, *baPWV* brachial-to-ankle pulse wave velocity**;** *hfPWV*, heart-to-femoral pulse wave velocity; *DM* diabetes mellitus, *HTN* hypertension, *BMI* body mass index, *SBP* systolic blood pressure, *eGFR* estimated glomerular filtration rate by CKD-EPI creatinine equation, *Ca* calcium, *FGF23* fibroblast growth factor 23

## Discussion

The kidney is the principal organ for production of klotho, and CKD is known to be associated with a klotho-deficiency state. CKD patients suffer from a high burden of CVD. In the present study, the serum klotho level was lower in advanced CKD stages. Klotho exhibited an independent negative association with LVMI. However, there was no significant association between klotho and PWV after adjustment in our subjects. Abdominal aorta calcification and coronary artery calcification were not significantly different among the klotho quartile groups. No differences in systolic or diastolic heart dysfunction were observed across klotho quartiles.

Previous studies showed that CKD patients were more likely to have cardiac structural changes in the absence of decreased LV ejection fraction [34, 35]. Lower prevalence of systolic and diastolic heart dysfunction was not surprising, given the exclusion of subjects with severe heart failure (New York Heart Association Class III or IV) from enrollment in the present study.

Xieet al. [12] showed that klotho-deficient CKD mice have aggravated cardiac hypertrophy and cardiac fibrosis compared with wild-type CKD mice. Intravenous delivery of a transgene encoding soluble klotho attenuated cardiac hypertrophy in the klotho-deficient CKD mice. The authors explained that downregulation of the stress-induced transient receptor potential canonical 6 (TRPC6)-mediated gene amplification loop by soluble klotho may play a role in the cardioprotection of uremic hearts [36].Yang et al.[16]reported that klotho protects against indoxyl sulphate-induced cardiac hypertrophy in CKD mice. They also showed that serum klotho levels are associated with the development of LVH in patients with CKD. They did not show an association between klotho and LVMI for CKD patients in an adjustment model. Most animal study results have suggested that klotho deficiency is associated with cardiac hypertrophy. However, clinical studies have shown mixed results with regards to serum klotho and cardiac hypertrophy. Tanaka et al. [37] reported that the lowest klotho tertile was associated with LV hypertrophy and systolic dysfunction only among patients with CKD stage G3a and G3b, respectively. Buiten et al. [17] showed that serum klotho was not independently associated with CVD, including LVMI, among 127 dialysis patients. This study was conducted with a small number of dialysis patients compared to our study. They also described that the association of soluble klotho with cardiovascular parameters might be diminished since the patients already developed end stage renal disease [17]. Seiler *et al.* [38] showed that soluble klotho was not significantly associated with cardiovascular outcomes for 444 patients with CKD stage 2–4. They did not assess each of cardiovascular parameters and mean eGFR was lower than that of our study subjects (45 ± 16 vs. 53.0 ± 30.7 ml/min/1.73m^2^). Our study included all stages of CKD patients and presented that klotho could be a marker of LVMI in predialysis CKD patients. The reason for the discrepancies between those studies and ours remains uncertain. However, there are possible explanations. First, these studies differed in race, kidney function, and number of subjects. Second, we examined cardiovascular parameters, rather than cardiovascular outcomes. Thirdly, patients with severe heart failure (New York Heart Association Class III or IV) were excluded in our study. However, the present study has a much greater statistical power, due to a larger number of CKD subjects analyzed. Soluble klotho plays important roles in anti-aging, anti-oxidant, and anti-vascular calcification [39],and CKD as a klotho-deficient state may have a close association with chronic cardiovascular complications. The present study showed the association of klotho with LVMI in a large number of CKD patients, with adjustment for markers of mineral bone metabolism such as phosphorous and calcium.

In an experimental study, *klotho* gene delivery into skeletal muscle inhibited medial hypertrophy of the aorta in an animal model of atherosclerotic disease [40], and klotho deficiency–induced arterial stiffening was mediated by upregulation of aldosterone levels [14]. Soluble klotho protects endothelial integrity by regulating calcium entry into vascular endothelial cells [40, 41]. Kitagawa et al. [42] reported that the serum klotho level was a significant determinant of arterial stiffness, defined as baPWV ≥1400 cm/s in 114 CKD patients. They showed a significant association only at baPWV ≥1400 cm/s. In our study, we also analyzed hfPWV as a central arterial stiffness marker. In another clinical study, arterial stiffness measured by baPWV increased in 109 CKD patients, but it was not related to klotho [43]. This study was performed only with a small number of diabetic CKD patients. Thus, there have been discrepancies among clinical study results. Further studies are needed to elucidate the association between klotho and arterial stiffness in CKD patients. This study has several limitations. First, owing to the cross-sectional nature of the study, it is hard to demonstrate the cause-effect inferences about the relationship between serum klotho levels and cardiac hypertrophy or arterial stiffness. Second, serum klotho has a circadian variation, meaning that examination at a fixed time is necessary [44]. Thirdly, we measured c-terminal FGF23 in this study. Lack of agreement between c-terminal and intact FGF23 measurements and also differences in their association with other biochemical parameters have been reported [45]. However, both the higher c-terminal and intact FGF23 values have been associated with increased mortality and poor outcomes in CKD patients.

## Conclusions

Serum klotho was an independent biomarker of LVMI but not arterial stiffness and vascular calcification. Further studies are warranted to elucidate the clinico-pathogenic significance of klotho for cardiovascular parameters, and whether any interventions to maintain or increase the serum klotho level can prevent cardiovascular events and mortality in CKD patients.

## Abbreviations

AAC: abdominal aorta calcification
ANOVA: one-way analysis of variance
BMI: body mass index
CACS: coronary artery calcium score
CI: confidence interval
CKD: chronic kidney disease
CKD-EPI: Chronic Kidney Disease Epidemiology Collaboration
CRP: C-reactive protein
CVD: cardiovascular disease
DM: diabetes mellitus
eGFR: estimated glomerular filtration rate
ELISA: enzyme-linked immunosorbent assay
HTN: hypertension; IDMS: isotope dilution mass spectrometry
iPTH: intact parathyroid hormone
IQR: interquartile range
KNOW-CKD: KoreaN Cohort Study for Outcome in Patients With Chronic Kidney Disease
LV: left ventricular
LVH: left ventricular hypertrophy
LVIDd: left ventricular internal diameter at end diastole
LVMI: left ventricular mass index
PWTd: posterior wall thickness at end diastole
PWV: pulse wave velocity
RWV: relative wall thickness
SD: standard deviation
SWTd: septal wall thickness at end diastole
VitD: vitamin D
